# Limitations of Online Information on Abdominal Aortic Aneurysm

**DOI:** 10.1155/2010/789198

**Published:** 2011-01-24

**Authors:** Carolyn G. Goldberg, Loren Berman, Richard J. Gusberg

**Affiliations:** Department of Surgery, Yale University School of Medicine, 333 Cedar Street, BB 204D, New Haven, CT 06510, USA

## Abstract

*Background*. Patients with AAA face a complex decision, and knowledge of the risks and benefits of each treatment option is essential to informed decision-making. Here we assess the current information on the internet accessible to patients regarding the management of AAA. *Study Design*. We performed a search on Google using the keywords “abdominal aortic aneurysm” and reviewed the top 50 web sites. We focused on information related to treatment options and alternatives to treatment and the risks of each option. *Results*. Twenty-seven websites were included in the study. Nearly 30% of websites discussed the risk of mortality and myocardial infarction after open surgery, compared to only 7.4% for both risks after EVAR. Other complications were listed by fewer websites. Fifty-five percent of websites reported that patients had a faster recovery following EVAR, but only 18.5% mentioned the risk of reintervention after EVAR or the need for long-term surveillance with CT scans. *Conclusions*. While most websites included descriptive information on AAA and mentioned the potential treatment options available to patients, the discussion of the risks of open surgery and EVAR was inadequate. These results suggest that websites frequently accessed by patients lack important information regarding surgical risk.

## 1. Introduction

When diagnosed with an abdominal aortic aneurysm (AAA), patients are confronted with a complex decision: whether or not to undergo invasive treatment for an asymptomatic but potentially fatal condition. If patients elect to pursue surgical treatment, many must further decide between open surgery and endovascular repair (EVAR). Knowledge of the natural history of AAA as well as the risks and benefits of all treatment options is essential for patients to make a well-informed decision. Effective communication of this information during the informed consent process can be challenging because of the volume and complexity of the information involved as well as the emotional impact of the potentially life-changing alternatives being considered. We have previously reported that many patients neither appreciated the scope of their options before AAA repair nor felt adequately informed prior to making a decision [1]. Given the complexity and variability of information, it is not surprising that many patients do not feel well informed about possible outcomes of surgical intervention. It has been well documented that education increases patient satisfaction with decision making [2–5]. 

The internet has the potential to provide patients with information and support as an adjunct to direct communication with the surgeon [6–8]. There are several advantages to the internet as a medium for patient information: it is widely used, easily accessible, and inexpensive. In 2008, it is estimated that 150 million Americans looked online to obtain health care information across health care fields [9]. This equates to over two thirds of all adults and 81% of all of those who go online [9]. The internet has the potential to supplement patient information, increase patient understanding of treatment options, and enrich the quality of both the initial dialogue with primary care providers as well as informed-consent discussions with surgeons. However, the assumption that an internet-informed patient is necessarily well informed could limit and/or adversely affect such discussions.

Despite the widespread use of the internet as a health care resource, many studies have found health-related information on the world wide web to be unreliable, outdated, or incomplete [10–16]. Few studies have systematically evaluated the information available to AAA patients online. Our study aims to quantify what information is immediately available on the internet to patients seeking to understand the diagnosis and treatment options for AAA. 

## 2. Methods

We simulated a patient search using the Google search engine with the keywords “abdominal aortic aneurysm.” Google was chosen because it is the most frequently used internet search engine among US home and work-web users [17]. We selected these keywords because this is the most natural phrase a patient newly diagnosed with AAA might enter into the search engine to learn more about the diagnosis. We did not limit or refine the search in order to simulate the method that a patient who was inexperienced at searching for health information might employ. We evaluated the first 50 websites generated by the Google search engine.

We also reviewed sponsored websites obtained from the “Sponsored Links” generated by an Ad Search engine. These websites appear at the top of the Google search page and in the margins. Known as mobile ads, these websites arise from sponsors that pay for placement on the same page as Google search results [18]. The first 15 sponsored websites were reviewed over three days (August 14–17, 2009.) The potential for commercial interest to influence content of information has been recognized as a barrier to patients obtaining unbiased information from the internet [19]. We wanted to determine if the type of information presented by the sponsored websites differed from that of the nonsponsored websites. 

 We included only websites that were immediately accessible to patients. We did not evaluate websites composed of links to other websites, duplicate sites, links to abstracts or full texts of scientific papers. We excluded websites that did not present clinical information for patients diagnosed with AAA, such as websites providing information about screening only. If a website had restricted access it was not reviewed. Data was collected from a website review over a one-week period from August 6–13, 2009. A repeat review performed in December of 2010 showed minimal changes.

We evaluated the completeness of information available to patients concerning treatment options, focusing on what we believed would be most directly relevant to decision-making. We recorded information in the following categories: (1) description of AAA, (2) treatment options and alternatives (including watchful waiting using serial imaging and nonintervention reflecting a decision opposing any AAA treatment), and (3) risks and benefits of each management option. We also evaluated basic characteristics of the websites such as whether they were specific to AAA repair only, whether they contained advertisements or informational videos, and if scientific articles or textbooks were listed as references.

Within the descriptive information category, we recorded whether a website included a definition of AAA, the symptoms of AAA, risk factors for developing AAA, tests used to diagnose AAA, the outcome of aneurysm rupture, and the risk of peripheral embolization. In addition, we recorded whether websites quantified the mortality of AAA without treatment and the mortality of a ruptured AAA.

For treatment options and alternatives, we noted whether websites described the indications for AAA repair, included EVAR and open surgery as options, and reported that only certain patients are eligible for EVAR. We also recorded whether websites mentioned “watchful waiting”—periodic surveillance with imaging of a known AAA that did not meet criteria for surgical intervention. This option differed from the option of nonintervention, defined as not undergoing surgery despite having an AAA that met size criteria for surgical intervention.

The criteria used for evaluating the risks and benefits described for each treatment was developed from responses to a national survey of vascular surgeons that identified which risks are essential to discuss during informed consent discussions for AAA repair [20]. For both EVAR and open repair, we included the risks of mortality, myocardial infarction, and renal failure. For open surgery, we included prolonged mechanical ventilation, and for EVAR we included the risks related to long-term endograft surveillance, reintervention, and postoperative rupture.

Information was marked as present if terms or synonyms were explicitly included in the text (e.g., risk of mortality was marked as mentioned if the terms “mortality” or “risk of death” were used but not if the term “safety” was used). When quantified, information on risk was evaluated for accuracy based on the range of values reported in the literature [20]. In addition, the text of the sites was examined for grossly inaccurate information. 

## 3. Results

Of the 50 websites evaluated in our search, 34 were nonredundant, accessible to the public and did not consist of a journal article or abstract. Seven of these websites were excluded for the following reasons. Three contained information on AAA screening recommendations only, one consisted of a CT scan of an AAA without text, one was an advertisement for Statins, one was an advertisement for an AAA textbook, and one consisted of a technical description of CT volumetric analysis used to diagnose an AAA. Most relevant websites were found between websites 1 through 20. There was a steep drop in relevancy after the first twenty websites, with few relevant websites found between websites 30 and 50. 

Seven percent of websites were specific to AAA repair alone, and 18% contained an informational video. One third of the websites contained advertisements. Thirty-seven percent of websites referenced at least one specific scientific article or textbook.

### 3.1. Descriptive Information about Abdominal Aortic Aneurysm (Table 1)

All 27 websites that were included in our evaluation defined AAA. These definitions ranged from a technical anatomical description to more patient-friendly information with teaching diagrams. Most of the websites also described symptoms of AAA (81%), the tests used to diagnose AAA (89%), and risk factors for developing AAA (96%).

Fewer websites quantified the chance of rupture for AAA without treatment (30%,) or the mortality after rupture (48% of sites).

### 3.2. Treatment Options and Alternatives (Table 2)

Most websites provided information about treatment options and alternatives, and indications for considering surgery. Nearly half of the sites reported information on what determines whether a patient is a candidate for EVAR. Although most websites described watchful waiting as a treatment option, fewer websites described the option of no surgical treatment once the aneurysm reached a size that would generally warrant surgical intervention. 

### 3.3. Risk and Benefits of Treatment Options (Table 3)

While 85% of websites described open surgery and EVAR treatment options, not all websites reported the risks related to these options. Mortality and myocardial infarction, the most commonly reported complications, were mentioned by only 29.6% of websites in the context of open surgery and 7.4% in the context of EVAR. Fewer websites described other complications after open surgery or EVAR. Only 18.5% and 7.4% of websites quantified the risk of mortality from open surgery and EVAR, respectively, and fewer websites quantified the probability of other complications occurring.

### 3.4. Information on EVAR As an Alternative to Open Surgery

Information provided on EVAR as an alternative to open surgery was found to be highly inconsistent. While 55% of websites reported that EVAR patients experienced a faster recovery than open repair and 44% of websites reported the decreased hospital stay compared to open surgery, only 18.5% of websites mentioned the risk of reintervention after EVAR, and only 40.7% described the need for long-term surveillance after EVAR. Although less invasive methods of monitoring may be used, CT scans are often performed annually to monitor patients after EVAR. None of the websites mentioned the risk from radiation or contrast exposure associated with annual CT scans, and none mentioned the persistent, though small, risk of AAA rupture after EVAR. Forty-four percent of sites did report that EVAR has not been evaluated in the long term.

### 3.5. Accuracy of Information Presented

In general, the information presented was accurate, and when complication rates were provided, they fell within the range of values that are accepted in the current literature [20]. There were a few instances, however, where websites provided grossly inaccurate information. For example, one website contained this statement: “Even patients who do not have symptoms from their AAAs require surgical intervention because the result of medical management in this population is a mortality rate of 100% over time due to rupture” (see Table 4 row (19)). Such comments were, however, a rarity.

### 3.6. Sponsored Websites

Of the 15 sponsored websites reviewed, 10 were eliminated because they were redundant or irrelevant to AAA patients as previously defined in the methods section. Three of the five websites evaluated were sponsored by companies that manufacture endografts, and the remaining two websites were sponsored by a university hospital and the American Association of Retired Persons. 

 Most websites presented descriptive information about AAA and presented surgical treatment options. Like the nonsponsored websites, there was a lack of information about risk. In fact, only one sponsored website reported that risks for open surgery include death, myocardial infarction, renal failure, prolonged mechanical ventilation, or impotence. None of the sponsored sites mentioned these risks in relation to EVAR. Two of the 5 websites mentioned the possibility of further reintervention or rupture after EVAR. Four of the sponsored links reported a decreased recovery time, and 2 websites mentioned the decreased time in the hospital after EVAR compared to open surgery. Two of the websites described the need for additional followup, and none mentioned the need for annual CT scans.

## 4. Discussion

Most websites included descriptive information on the physiology and anatomy of AAA and mentioned the surgical treatment options available to patients. However, only one-third of websites presented nonintervention as an option for patients. Underreporting of this option may mislead patients into believing that they must have surgery, irrespective of comorbidities and other factors, when in fact nonintervention is a reasonable option for certain patients [21, 22].

When considering the surgical options, the discussion of the risks of open surgery and EVAR was inadequate. The most common risk reported was mortality, which was reported by less than one-third of sites as a risk for open repair and less than 10% of sites as a risk for EVAR. The lack of information on risks may lead patients to form unrealistic expectations of treatment outcomes. Within the discussion of risks, few sites quantified the probability that a complication would occur. When there is no information about the probability of a complication occurring, it is difficult for patients to understand the severity of the risk [4].

When EVAR was presented as an alternative to open surgery, we found that websites tended to present more information about the benefits and less information about the risks of EVAR. Over half of websites reported decreased length of hospital stay after EVAR compared to open repair. In comparison, less than 20% reported a risk of reintervention after EVAR. Although 40% of sites alluded to the concept of increased follow-up after EVAR, only 18.5% specified the need for annual CT scans. In addition, 17.4% of websites described possible immediate postoperative complications after EVAR. 

Reporting of surgical risk was even lower among industry-sponsored websites. Only one such website reported any surgical risks for open surgery, and none of the sponsored websites reported the risk of death, myocardial infarction, renal failure, or impotence in relation to EVAR. Of note, three out of the 5 websites that we evaluated were sponsored by industries that produce equipment for EVAR. It is possible that those industry-sponsored websites would underrepresent the disadvantages of EVAR, as there are obvious financial incentives for promoting this treatment option. Our observation that 4 out of the 5 sponsored websites described EVAR as having a shorter recovery time than open surgery is consistent with the premise that these sites may be more likely to emphasize the advantages rather than disadvantages of EVAR. These websites did not, however, report the risks of open surgery so it is possible that the lack of surgical risk information was simply consistent with the overall trend that we observed for the nonsponsored websites. More industry-sponsored links would need to be reviewed to draw a definitive conclusion on the type of information offered.

Other studies have suggested that websites tend to underemphasize the potential complications of surgical treatment. In a review of thyroidcancer information available to patients online, Air et al. found that websites contained a substantial amount of descriptive information on anatomy and physiology of thyroid cancer, but lacked information on thyroidectomy including descriptions of perioperative aspects of care and the risks of surgery [11]. Smart and Burling found a similar trend for online information for patients undergoing interventional radiology procedures. Nearly 80% of the websites described preparation for surgery and care after surgery, while only 21% of websites described any risks of the procedure [16].

Few studies have systematically evaluated the information available to patients for AAA on the internet. In 1999, Soot et al. determined that patient-oriented information for common vascular diseases is difficult to find on the internet, in part due to the large number of irrelevant sites. Although information on the proportion of websites that included risks of surgical procedures was not published, the study found that 67% of sites were not oriented to patient education and did not enhance patient knowledge [13]. Our findings confirm that over the last 10 years, it has remained a challenge for patients to obtain essential treatment information on the internet.

Despite a few examples of misleading or incorrect statements, websites did generally provide accurate, though incomplete, information. The few websites that quantified probabilities of post-operative complications did fall within the range of those reported in a national survey of vascular surgeons during informed consent discussions for AAA repair [20]. However, few websites reported the information considered by the majority of surgeons to be integral to an informed consent process. It was this overall lack of information or inconsistent information about risks and benefits of EVAR versus open repair that was misleading rather than the provision of inaccurate information.

Our study has several limitations. First, our compilation of information is likely to overestimate the amount of information that most patients would obtain online. Several websites contained vocabulary and diagrams that appeared to be targeted at physicians and might be challenging for patients without a medical background to interpret. In addition, as has been mentioned in other reviews of internet health care information, our results represent information available during a brief time period [8]. Because the internet is constantly changing, the 50 websites that we evaluated may not remain in the top 50 websites encountered on a Google search, and the information within websites is also dynamic. Finally, we did not observe patients actually navigating the internet, and it is possible that the pattern of searching would be different from our simulated pattern. For example, it is possible that patients would only review the first few websites produced by a Google search and therefore would obtain variable information depending on which websites were encountered. One study evaluating how search engine users search for results for a research query found that most users did not view more than three results on a page and few viewed the results in the order of their ranking [23]. Furthermore, our evaluation was restricted to a Google search, and we recognize that there are a variety of other search engines available.

Our results suggest that websites frequently accessed by patients lack important information regarding surgical risk. It has long been recognized that the quality of information online is variable [7]. In order to combat some of this variability, the American Medical Association (AMA) has developed principles to guide development and posting of website content [19]. These guidelines are meant to aid the development and maintenance of AMA websites but do not ensure that the available information is sufficient. We found that it was rare for websites to fully and reliably meet the needs of patients seeking information about the diagnosis and treatment of AAA.

The lack of information available to patients has important implications for the physician-patient encounter. Even proactive patients who are seemingly wellinformed may lack important information about health risk. Patients who form treatment preferences based on information obtained online are likely to have based these preferences on incomplete and possibly misleading information about the treatment options and their risks, potentially leaving them with unrealistic expectations of outcomes. Assessing the degree to which patients are making informed choices requires an understanding of the adequacy of information obtained from the internet and patients' ability to interpret this information. Surgeons and other physicians who regularly discuss AAA management options with their patients should be aware of the potential for patients to arrive with preconceived notions based on what they have read online and be prepared to provide more complete information on risk and outcomes. Efforts should also be made to improve the quality of information available on the internet for patients with AAA.

## Figures and Tables

**Table 1:** Descriptive information.

Information reported	Number (%)
Definition of AAA	27 (100)
Symptoms of AAA	22 (81)
Tests used to diagnose AAA	24 (89)
Risk of peripheral embolization	10 (37)
Risk factors for developing AAA	26 (96)
Describe outcome of rupture	19 (70)
Quantify mortality of aneurysm rupture without treatment	13 (48)
Quantify risk of mortality from rupture	8 (30)

**Table 2:** Treatment options and alternatives.

Information reported	Number (%)
Indications for surgery	24 (89)
Description of EVAR and open treatment options	23 (85)
Information reported on what determines if patient is a candidate for open or EVAR	13 (48)
Watchful waiting (periodic surveillance of an aneurysm that does not meet criteria for surgical treatment)	25 (93)
Nonintervention for aneurysm that meets criteria for surgical treatment	9 (33)

**Table 3:** Risk and benefits of treatment options.

Risks defined as essential to discuss based on national survey of vascular surgeons:	No. (percent) of websites to report risk		No. (percent) of websites to quantify risk	
	Open	EVAR	Open	EVAR
Mortality	8 (29.6)	2 (7.4)	5 (18.5)	2 (7.4)
MI	8 (29.6)	2 (7.4)	1 (3.7)	0 (0)
Prolonged mechanical ventilation (open only)	1 (3.7)	NA	0 (0)	NA
Renal failure	5 (18.5)	1 (3.7)	0 (0)	0 (0)
Impotence	3 (11.1)	0 (0.0)	1 (3.7)	0 (0)
Reintervention rate (EVAR only)	NA	5 (18.5)	NA	1 (3.7)
Post-operative rupture rate (EVAR only)	NA	2 (7.4)	NA	1 (3.7)

**Table 4:** Evaluated websites.

(1)	MedicineNet.com	http://www.medicinenet.com/abdominal_aortic_aneurysm/article.htm
(2)	medlineplus: medical encyclopedia. NIH and National Library of Medicine	http://www.nlm.nih.gov/medlineplus/ency/article/000162.htm
(3)	MedlinePlus. Tutorials.x-plain: patient education institute	http://www.nlm.nih.gov/medlineplus/tutorials/abdominalaorticaneurysm/htm/index.htm
(4)	Society for Vascular Surgery. VascularWeb	http://www.vascularweb.org/vascularhealth/Pages/abdominal-aortic-aneurysm.aspx
(5)	Society of Interventional Radiology (SIRS)	http://www.sirweb.org/patients/abdominal-aortic-aneurysms/
(6)	Emedicine from WebMD	http://emedicine.medscape.com/article/463354-overview
(7)	Collaborative Hypertext of Radiology: quick reference for physicians and medical students	http://chorus.rad.mcw.edu/doc/00990.html
(8)	Wikipedia	http://en.wikipedia.org/wiki/Abdominal_aortic_aneurysm
(9)	WebMD healthwise	http://www.webmd.com/heart-disease/tc/aortic-aneurysm-overview
(10)	Keck School of Medicine, USC. USC center for vascular care	http://www.surgery.usc.edu/divisions/vas/abdominalaorticaneurysm.html
(11)	Mayo Clinic	http://www.mayoclinic.org/aortic-aneurysm/
(12)	familydoctor.org	http://familydoctor.org/online/famdocen/home/articles/883.html
(13)	Society of Thoracic Surgeons	http://www.sts.org/patient-information/aneurysm-surgery/aortic-aneurysms/
(14)	Mayo Clinic (.com not.org, as was on previous website.)	http://www.mayoclinic.com/health/aortic-aneurysm/DS00017/DSECTION=tests-and-diagnosis
(15)	USA Today	http://www.healthscout.com/ency/68/447/main.html
(16)	Univ. Maryland Medical Center	http://www.umm.edu/vascular/aaa.htm
(17)	Penn State	http://www.hmc.psu.edu/healthinfo/a/abaortic.htm
(18)	Up to date for patients	http://www.uptodate.com/patients/content/topic.do?topicKey=~J.RRzC2BSKg6J
(19)	EMedicine Specialties > Radiology > Vascular/Interventional	http://emedicine.medscape.com/article/463354-overview
(20)	Baylor College of Medicine Dept of Surgery	http://www.debakeydepartmentofsurgery.org/home/content.cfm?content_id=274&proc_name=Abdominal+Aortic+Aneurysm
(21)	Surgical Care Associates (private surgical group in Louisville, Kentucky 40207)	http://www.aorticaneurysm.com/
(22)	WCVBTV/DT Boston abc.: Q&A with doctor from the Beth Israel Deaconess Medical Center	http://www.thebostonchannel.com/bethisrael/18469885/detail.html
(23)	Cochrane Collaboration	http://www.cochrane.org/reviews/en/ab002945.html
(24)	Stanford Hospital and Clinics medical center	http://www.stanfordhospital.com/clinicsmedServices/COE/surgicalServices/vascularSurgery/patientEducation/abdomin
(25)	Medlineplus: Sponsored by GORE excluder endoprosthesis.	http://www.or-live.com/distributors/nlm-flash/wlg_1903/rnh.cfm?id=675
(26)	Emedicine for Patients (Emedicine Health)	http://www.emedicinehealth.com/aortic_aneurysm/article_em.htm
(27)	Chiropractic Site	http://www.chiroweb.com/mpacms/dc/article.php?id=39225
